# Evaluation of MetriGenix custom 4D™ arrays applied for detection of breast cancer subtypes

**DOI:** 10.1186/1471-2407-6-59

**Published:** 2006-03-15

**Authors:** Aslaug Aamodt Muggerud, Hilde Johnsen, Debra A Barnes, Adam Steel, Per Eystein Lønning, Bjørn Naume, Therese Sørlie, Anne-Lise Børresen-Dale

**Affiliations:** 1 Department of Genetics, Faculty division, The Norwegian Radium Hospital, University of Oslo, N-0310 Oslo, Norway; 2 MetriGenix Corporation, Toronto, ON, Canada; 3 Oncology, The Norwegian Radium Hospital, Oslo, Norway; 4 Department of Oncology, Haukeland Hospital, University of Bergen, Norway

## Abstract

**Background:**

Previously, a total of five breast cancer subtypes have been identified based on variation in gene expression patterns. These expression profiles were also shown to be associated with different prognostic value. In this study tumour samples from 27 breast cancer patients, previously subtyped by expression analysis using DNA microarrays, and four controls from normal breast tissue were included. A new MetriGenix 4D™ array proposed for diagnostic use was evaluated.

**Methods:**

We applied MetriGenix custom 4D™ arrays for the detection of previously defined molecular subtypes of breast cancer. MetriGenix 4D™ arrays have special features including probe immobilization in microchannels with chemiluminescence detection that enable shorter hybridization time.

**Results:**

The MetriGenix 4D™ array platform was evaluated with respect to both the accuracy in classifying the samples as well as the performance of the system itself. In a cross validation analysis using "Nearest Shrunken Centroid classifier" and the PAM software, 77% of the samples were classified correctly according to earlier classification results.

**Conclusion:**

The system shows potential for fast screening; however, improvements are needed.

## Background

A feature common to all commercialized or in-house DNA expression arrays up to date is the two-dimensional nature of the format. An array platform (MetriGenix 4D™ Array System, MetriGenix, Toronto, Ontario, Canada) was introduced in which molecular interactions occur within three-dimensional volumes of ordered microchannels rather than at a two-dimensional surface [1]. The microchannel geometry partition target solution into small volumes that enhance the mass transport of targets to probes, resulting in reduced hybridization times and provides greater binding capacity. The method described here utilizes the proprietary Universal Linkage system (ULS^®^) technology from Kreatech Biotechnology (Amsterdam, The Netherlands) for linking biotin to amplified RNA [2,3]. The probes are detected by single-colour chemiluminescence with high sensitivity [4]. MetriGenix developed a special designed hybridization station and detection unit to process custom 4D arrays.

Molecular signatures based on gene expression patterns of breast carcinomas, specifying different subtypes of tumours, have been identified by the use of DNA microarrays [5-7]. Specifically, the set of 552 "intrinsic" genes reported by Perou and Sorlie [6] was found to segregate breast cancers into 5 distinct groups based on gene expression profile. This profile has been validated in later studies and also shown to have prognostic value [5,7-9]. Two of the subtypes (luminal A and luminal B) belong to the estrogen receptor positive (ER+) group of tumours, while the tumours in the three other subgroups (basal-like, ERBB2+ and normal-like) in general, all are estrogen receptor negative (ER-). Interestingly, the estrogen receptor alpha and HER2/ERBB2, the major parameters characterizing 3 of the 5 different classes, are the goals for the most successful targeted therapies in breast cancer, underlining a fundamental role in biological control.

Gene expression patterns from 269 genes selected to optimally define the different subtypes of breast cancer were analyzed in breast tumours and normal tissue from 31 individuals (20 early breast carcinomas, 7 locally advanced breast carcinomas and 4 normal tissue samples). The aim of this pilot study was to investigate whether this type of arrays would have the potential as a diagnostic tool for molecular classification in a clinical setting.

Here we show that the MetriGenix 4D™ arrays with a limited number of carefully selected probes, perform similarly to other microarray platforms, although technical difficulties were experienced with the prototype system.

## Methods

### Tumour material and RNA extraction

In total, 27 breast tumour samples and 4 normal tissue samples were analyzed in this study. Among the tumour samples, 20 biopsy tissues from early breast carcinomas were included of which ten have been previously sub-classified as luminal subtypes, the other half as non-luminal subtypes using traditional two-dimensional microarray platforms such as Stanford cDNA arrays, Agilent Human Whole Genome Arrays and Applied Biosystems Human Genome Survey Microarrays [10]. Furthermore, tumour tissues from 7 locally advanced breast cancers were also included. These samples are part of a cohort of thirty-five patients, and have previously been described [11]. Three were classified as luminal subtypes and four as non-luminal subtypes [9]. In addition, control samples were taken from mastectomy specimens from four breast cancer patients. We selected tissue distant from the tumour, and verified that it consisted of unaffected breast tissue by HE (haematoxylin-eosin) stains of frozen sections. The scientific protocols (tissue sampling and laboratory analysis) of the samples were approved by the Regional Committee for Medical Ethics (health region II) for the M-samples (reference S- 97103) and Regional Committee for Medical Ethics (health region III) for the F-samples (reference 39/92–69.91).

Total RNA was extracted from fresh frozen tissue samples by using TRIzol^® ^(Invitrogen, Carlsbad, CA, USA) as described by the manufacturer. The RNA quality was evaluated by microcapillary electrophoresis using an Agilent 2100 Bioanalyzer (Agilent Technologies, Palo Alto, CA, USA) and concentration measured by using NanoDrop (NanoDrop Technologies, Wilmington, DE, USA).

### Selection of genes immobilized on the MetriGenix-Chip

We selected 269 genes that best represented the classification scheme in breast cancer to be synthesized and immobilized on the MetriGenix-Chip (see Additional file 1 for a complete listing of probes). The genes were selected from the intrinsic gene list as defined in Perou et al. 2000 [6] and Sorlie et al. 2001/2003 [8,9] by a semi-supervised method. A nearest shrunken centroid analysis using PAM was performed to reduce the number of genes in the classification scheme. Thus, the top 226 genes from this list were included in the 269 selected for syntheses. In addition, genes that distinguish lobular from ductal carcinomas [12], and cell cycle associated genes were added to the chip to enable other types of classification.

### MetriGenix-Chip-Preparation and hybridization

Total RNA was amplified using a two-step cRNA synthesis scheme typical for microarray experiments. First strand cDNA was synthesized by annealing T7-(T) 24- primer (100 pmol/μl) with 5 μg total RNA in a final volume of 12 μl at 70°C for 10 min, followed by addition of first-strand master mix (5× First Strand Buffer, 0.1 M DTT, 10 mM/each dNTPs mix, 25 U/μl RNaseOUT and 200 U/μl SuperScript II) to a final volume of 20 μl. The reaction was incubated at 42°C for one hour. Second strand synthesis followed immediately by adding 5× Second Strand Buffer, 10 mM/each dNTPs mix, 10 U/μl *E.coli *DNA Ligase, 10 U/μl *E.coli *DNA Polymerase I and 2 U/μl RNaseH to a final volume of 150 μl, and incubating at 16°C for two hours. To complete the reaction, 5 U/μl T4 DNA Polymerase were added and further incubated at 16°C for five min (all reagents supplied by Invitrogen). Double-stranded cDNA was purified in Phase-Lock Gel Tubes (Eppendorf AG, Hamburg, Germany) and *in vitro *transcribed by Ambion's MEGAscript ™ T7 High Yield Transcription kit (Ambion, Inc. Austin, TX, USA) followed by cleanup with RNeasy^® ^RNA isolation kit columns (Qiagen). Amplified cRNA was evaluated on the Agilent 2100 Bioanalyzer (Agilent Technologies). The cRNA was biotin-labelled using MetriGenix Bio ULS (universal linkage system) (0.5 Units/μl) in a one-step chemical coupling reaction at 85°C for 30 min (MetriGenix and KreaTech Biotechnology, Amsterdam, The Netherlands).

Prior to hybridization the biotin-labelled cRNA were mixed with spike-in controls (for hybridization quality), Sample Dilution Buffer 2 (MetriGenix) and herring sperm DNA (Invitrogen) and denatured for 5 min at 90°C. The sample was then injected into the sample compartment of the 4D array, along with blocking and staining reagents into their respective compartments.

Custom 4D arrays to monitor the genes of interest were supplied by MetriGenix (Baltimore, Maryland). For each gene, a 50- to 60-mer probe was designed based on publicly available sequences and to have GC content in the range of 45 to 55 percent and a melting temperature between 64 and 68°C. For product quality control (QC) the following steps were performed: First, hybridization was performed with just the complements to the control probes to confirm that there was no cross-contamination of probes on the chip. Second, a test cRNA was hybridized to the chip in the absence of the control targets; since the controls were bacterial and the test cRNA mammalian, no hybridization was observed in the control probes (otherwise the chips failed QC). Third, control targets were added to every cRNA that was hybridized to the chip and the intensity of the spots was used qualitatively to confirm the hybridization results. Probes were synthesized with a 5' amino modification and printed on MetriGenix arrays using a Gene Machine Omnigrid arrayer. The arrays are housed in a 4D cartridge that includes reagent reservoirs and interfaces with the MGX2000 and MGX1200CL array processing stations.

4D array hybridizations were performed on the MGX™ 2000 hybridization station, which controlled all subsequent steps (blocking and buffer flushes, hybridization time and temperature). After four hours of hybridization (3 h for hybridization to the corresponding probes and 1 h for blocking, washing and staining of the reactive spots with HRP-streptavidin), the chip was placed in the MGX 1200 CL detection unit for chemiluminescence (CL) detection with exposure times usually ranging from 2 to 5 s. Subsequent image analysis was performed with the MetriSoft software (MetriGenix) that generated an excel file containing the experiment data for subsequent analysis.

### Data analysis

The Metrisoft software operated on two different concepts, the noise floor and a stringent threshold value, to filter spots in the individual chip analysis. The noise floor was a value calculated by the software in each individual chip analysis that related to the amount of noise in the chip and which was subsequently used to determine the threshold value. The stringent threshold value was calculated as 3 times the noise floor, an empirical estimate of an 'absent' spot based on the image noise. Any signal below this value was not considered significant and assigned to the threshold value. For intra-chip normalization, the signal intensity of each individual spot was divided by the threshold to produce the normalized values within each chip. Data from 3 successful hybridized controls (one control with poor chip image was rejected from further analysis) were averaged for each gene to obtain a mean expression value. Next, to create log-transformed (base 2) pseudo ratios the value of each sample was divided by the mean of the three controls for every gene.

Principal Component Analysis (PCA), hierarchical clustering and ANOVA were performed by using Avadis Prophetic software (Strand Genomics, Bangalore, INDIA). Data were mean-centered, clustered using Euclidean distance measures and visualized using a heat map in which numeric values are represented in colour intensities (high levels in red, low levels in green). For ANOVA, samples were assigned class designations and gene expression data were analyzed assuming equal variance. Data were ranked based on p-values and F-statistics. In addition, a set of genes that best discriminated the two identified main subtypes of breast cancer were determined using "Nearest Shrunken Centroid classifier" and the PAM software [13]. For this analysis, pseudo ratios were generated using an average of all tumour samples as the denominator, to prevent the normal tissue samples from driving the analysis. PAM analysis was also performed with the pseudo ratios used for PCA, ANOVA and clustering analysis (see above) with similar results (data not shown).

## Results and discussion

The aim of this study was to demonstrate whether these novel arrays would have the potential to be used in molecular classification in a diagnostic setting in a future implementation. The limited number of MetriGenix chips available in this study allowed us to successfully hybridize and analyze altogether 25 of 31 samples (16 early breast carcinoma-, 6 locally advanced breast carcinoma- and 3 of the controls). Therefore, in the further evaluation with respect to the accuracy in classifying the samples we concentrated on the luminal (luminal A and B) vs. non-luminal (basal-like, ERBB2+ and normal-like) groups instead of all five subtypes.

### Statistical analysis of luminal versus non-luminal tumours

**Figure 1:**
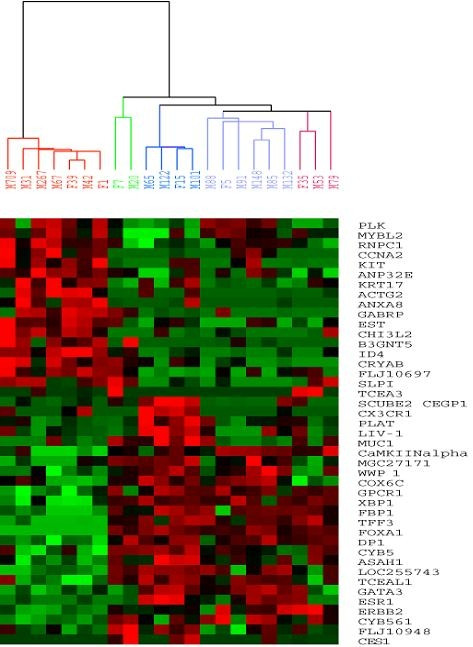
**Hierarchical clustering of luminal and non-luminal breast tumour samples**. The 43 most significant genes from the ANOVA analysis (luminal vs. non-luminal, p < 0.01), excluding the control samples, were used in a hierarchical clustering analysis. Coloured branches represent the different subtypes as previously determined using other DNA microarray platforms [9,10]: Dark blue = luminal A, light blue = luminal B, green = normal-like, red = basal-like and purple = ERBB2+. M = early breast cancer, F = locally advanced breast cancer.

To evaluate the data generated by the MetriGenix analyses, an ANOVA analysis was performed excluding the control samples to identify the genes that best separated the samples into two groups; luminal and non-luminal (see Additional file 2). Genes with a p < 0.01 (n = 43) were used in a hierarchical clustering and a clear separation of the luminal and non-luminal samples was seen (figure 1). The tumours previously classified as luminal A showed moderate to high expression of luminal epithelial specific genes including the *ESR1, GATA3*, *XBP1, MUC1 *while the basal-like tumours showed no expression of these genes. Further, the luminal cluster was divided into four sub-groups corresponding to the luminal A (dark blue), luminal B (light blue), the latter with less expression of luminal epithelial specific genes expressed, the previously identified ERBB2+ - (purple) and the normal-like group (green). Although there were few samples analyzed in this study, those representing each subtype grouped together.

To test whether the clustering pattern could be visualized by using data from all the 226 "intrinsic" only genes printed on the 4D array, a hierarchical clustering was performed (see Additional file 3). As previously noted, the basal-like tumours formed a distant group, whereas the remaining tumours clustered together on a separate branch.

**Figure 2:**
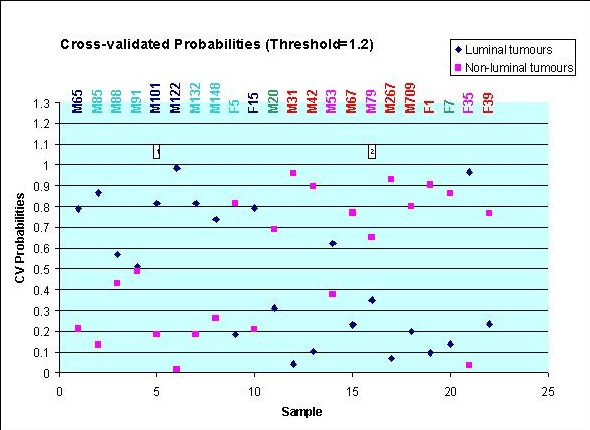
**Prediction of tumour subtypes using PAM**. Cross-validated probabilities are shown for luminal tumours (group 1, blue diamonds) and non-luminal tumours (group 2, pink squares). Coloured samples names represent the different subtypes previously determined using different microarray technologies: Dark blue = luminal A, light blue = luminal B, green = normal-like, red = basal-like and purple = ERBB2+. M = early breast cancer, F = locally advanced breast cancer.

To further evaluate the dataset, prediction of tumour subtypes was performed using the PAM software and the results are shown in figure 2. Using a threshold of 1.2, 27 genes were selected that were differential expressed between the two sample groups; luminal and non-luminal (see Additional file 4). Of these, 13 were also found significant (p < 0.01) in the ANOVA analysis (see Additional file 2). Ten-fold cross-validated probabilities were computed for luminal (group 1, blue diamonds) and non-luminal (group 2, pink squares) tumours. Samples M88 and M91 (both luminal B) were not classified since the CV probabilities was about 0.5. Furthermore, the luminal B sample F5 and the two ERBB2+ samples M53 and F35 were misclassified by the ten-fold cross-validation. One explanation may be that the luminal B expression profile have some common features both with the luminal A and with the basal-like expression profiles as seen in previous studies [6,8,9].

**Figure 3:**
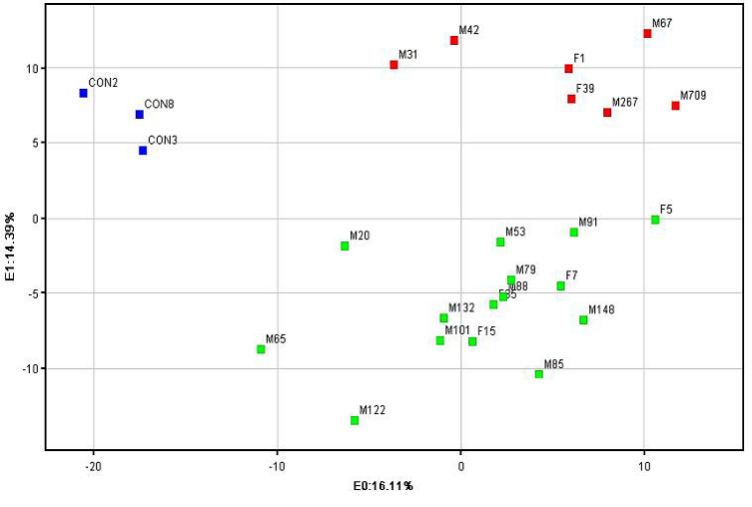
**Principal Component Analysis (PCA)**. Red squares indicate basal-like samples, green squares indicate all other samples (luminal A and B, ERBB2+ and normal-like) and blue squares indicate control samples.

Principal component analysis (PCA), based on all genes on the 4D array, confirmed the groupings of samples seen in the hierarchical clustering analysis (figure 3). Again, a major distinction of the basal-like samples was observed.

### Microarray platform performance

The sample preparation procedure including two-step cRNA synthesis and hybridization/detection was accomplished in three days. An advantage of the system was the short hybridization time of four hours; however, the multiple-day sample preparation protocol makes the overall analysis not much shorter than other microarray platforms. The system throughput was limited to a single chip per hybridization unit and the handling and purification was difficult due to small exterior buffer reservoirs and small buffer tubes, leading to contamination concerns. Moreover, it was difficult to trace errors, both on the hardware system and the Metrisoft software, as the system lacked an error message function. For more extensive applications, the image analysis software will require significant refinement. For example, artefacts such as pinspots and high background on the sample images occurred that led to the rejection of several arrays from further analysis. In addition, when analysing the images in the Metrisoft software, we consistently observed that the spot-finder did not find all of the spots on the image and manually re-reading of the image was necessary.

## Conclusion

We conclude that by selecting only the data from the well performed arrays (25/31) and by using key signature genes for breast-cancer subtypes immobilized on the MetriGenix 4D™ array, we were able to classify samples into the same subtypes as they were previously classified by using other microarray techniques, with a relatively high probability (23% misclassification). With higher throughput and improved performance this microarray platform has the potential to be a valuable tool for rapid routine gene expression profiling.

## Competing interests

DB and AS are previous employees of MetriGenix. MetriGenix is a private company that manufactured the arrays employed in this study, which was performed on a collaborative basis. All other authors declare that they have no competing interests.

## Authors' contributions

AM was involved in laboratory experiments, analyzing the data and drafted the manuscript. HJ was involved in laboratory experiments. DB carried out the statistical computation of the data. AS were involved in data analysis. PEL and BN were involved in providing breast cancer samples. TS and ALBD initiated and designed the study and were involved in writing the manuscript.

## Pre-publication history

The pre-publication history for this paper can be accessed here:



## Supplementary Material

Additional File 1 **Genes-on-chip**. A listing of the genes immobilized on the 4D array along with sequence information of each probe. Click here for file

Additional File 2 **ANOVA-analysis output**. ANOVA analysis of all the genes immobilized on the MetriGenix breast cancer chip. Click here for file

Additional File 3 **Hierarchical clustering of "intrinsic" only genes**. Hierarchical clustering diagram of the 226 "intrinsic" only gene subset immobilized on the MetriGenix chip. Coloured branches represent the different subtypes as previously determined using a different DNA microarray platform [10]; Dark blue = luminal A, light blue = luminal B, green = normal-like, red = basal-like and purple = ERBB2+. M = early breast cancer, F = locally advanced breast cancer. Click here for file

Additional File 4 **PAM-analysis output**. PAM output of the most differentially expressed genes between the two groups: luminal and non-luminal. Click here for file
