# Supplementary material for: Evaluation of MetriGenix custom 4D™ arrays applied for detection of breast cancer subtypes
Source: BMC Cancer. 2006 Mar 15;6:59. doi: 10.1186/1471-2407-6-59 (PMC1421426; doi:10.1186/1471-2407-6-59)
Supplement: Additional File 1 — Genes-on-chip. A listing of the genes immobilized on the 4D array along with sequence information of each probe. [file 1471-2407-6-59-S1.pdf]

Supplementary table 1
Genes immobilized on the MetriGenix breast cancer chip

The genes MGRN1 and TLE3 are immobilized twice to the chip with two different probes.

Gene Symbol	Accession Number	Sequence
ACADSB	NM_001609	GCTGTAACCAGCATGTCAAATGTGAAGATTATTGTGTGGGATCCTTTGGT
ACOX2	NM_003500	TGGAGATCCAAGCTATGAAATAACCAACAGTATTCAAGAAGCAACCAGCA
ACTG2	NM_001615	TTCTCCAAGGATCCCCTCGAGACTACTCTGTTACCAGTCATGAAACATTA
ADRA2A	NM_000681	CATCTGCATTTCTACATGTTTTAGACAGAGACAATTTAAGGCCTGCACTC
ADRM1	NM_007002	AGAAAGAGGGCGACACGAAGGACAAGAAGGACGAAGAGGAGGACATGAGC
AGTR1	NM_031850	TGCTGCACTGGTCCCAAGTAGTAGTGTCCTCCTAGTATATTAGTTTGATT
AKR1C3	NM_003739	CGACTTCAGTCAACTACAGCTGAGTCCATAGGCCAGAAAGACAATAAAT
ALCAM	NM_001627	ATTGCTATCAGCAATGCCCCAAACTTTCTCATAAGCACCTAAAACCCAA
ALDH1A2	NM_003888	AAGTGTTAGCTTCCTTTCCTTCTTCCATCCCTTCTTCCATCTTTCCAGCT
ANP32E	NM_030920	ACACTTAGAGCCTCCAGATAACTTTTAAGACTTATTTAGCTTTGTGGGTGG
ANXA1	NM_000700	AAGCTTCATCAAGCCATGAAAGGTGTTGGAACTCGCCATAAGGCATTGAT
ANXA8	NM_001630	TGGGTACCTGGAGATTCTGAAGTGCCTTTGCTGTGGTTTTCAAAATAA
ANXA9	NM_003568	AGCACCACATTCTCTAGCTTCTTGTTGAGGCTGGAACTGTTTCTTTAA
AOC3	NM_003734	AGGTCTGGATTGGGGATGGGGACAAAGAAATAGCAAGAGATGAGAAACAA
APEG1	NM_005876	TTGACAAAGGCCTTACCAGGAAGGGTTAGGACACTGACCATTCTAGAAAT
APM2	NM_006829	CCTCCCAAATTCGGAAATCCAATCCAACGGTCTCAGGAATGTTTTCCAT
APOD	NM_001647	CTGACTTCTAATAACATTGATGTCAAGAAAATGACGGTCACAGACCAGGT
ARPC5	NM_005717	ATATCGAGTAGCTCTAAAACAAACCACCTGACCAAGAGGGAAGTGAGCTT
ASAH1	NM_004315	TGTGCAAGACCGTTTGTCCACTTCATTTTGTATAATCACAGTTGTGTTCC
ATP1A2	NM_000702	TACTTTTCAGGCACTTTAGAAATATCCTTTTAGAAGCAGCGAGTGCATGG
ATP6V1G1	NM_004888	ATTCTTGCTAATGGTTAGAACAGTGAATACTAGTGGAATTGTTTGGGCTGC
B3GNT5	NM_032047	AAGGTTGCCATTGGTTGAAAACATAAGTGTCTCTGGCCATCAAAGTGATC
BCL2	NM_000633	CCCTCCAAGGTAGAATTTGCAAGAGTGACAGTGGATTGCATTTCTTTTGG
BECN1	NM_003766	ATACTGAATGCCGACTCTGCTCTGTGTTAGAGATATGAAATGGTGTTTGA
BF	NM_001710	CCCTTGATAGTTCACAAGAGAAGTCGTTTCATTCAAGTTGGTGTAATCAG
BLVRA	NM_000712	ACTTCCAAGATGGCACCAGCATTTGGTTCTTCTCAAGAGTTGACCATTAT
BMP4	NM_001202	ACTCACCCACACACTACACAGACTGCTTCCTTATAGCTGGACTTTTATT
BPHL	NM_004332	GTTGCTGCCTGTTAACATGATGCCTTTGAAACTCTCCGCCTTTGAAACTT
BTG3	NM_006806	TAGAAGTGGACGATTTGTTCTAGCACCTTTGAGAATTTACTTTATGGAGCG
C10orf7	NM_006023	GCTCACAAGCTAATAGACTTCCTTAAGCTGAAGAGAAATCAGCAGGAGGA
C14orf132	NM_020215	CCACGTGCCCAGTCCTATTTCTGCCAGTTGAAGGCATACTAATATTCTTTA
C4A	NM_007293	CTGGAGAAGCTGACCTCCCTCTCTGACCGTTACGTGAGTCACTTTGAGAC
CACNA2D2	NM_006030	ATTAATGTCATGTTGCTTGTCGCTTTCTCAGTGTGTGTGTGTGGTCCATG
CaMKIINalpha	NM_018584	TTACTAGCTTGCTACCCACGATGAAATCAACAACCTGTATCTGGTATCAG
CAPN6	NM_014289	GTCATTATGAAATGTACTTCCCTCCATCTTAACCTATCAACTTTCTGCCC
CAV1	NM_001753	CATGTCTGTTCTACATAGATGCTTAGTCCCTCATGCAAATCAATTACTGG
CBR1	NM_001757	AGCACTCACTAATGTACTACTAATTGAGCAACCTACGCACTCAGTTGACT
CCNA2	NM_001237	AGACCTACCTCAAAGCACCACAGCATGCACAACAGTCAATAAGAGAAAAG
CCND1	NM_053056	TACCTGTAGGACTCTCATTCGGGATGATTGGAATAGCTTCTGGAATTTGT
CCNE1	NM_001238	CCCTCTCCACATTATCAGTTGACAGTGTACAATGCCTTTGATGAACTGTT
CCNE2	NM_057749	TCCATTGTGAGATAAGGACAGTGTCAAAGTGATAAAGCTTAACACTTGACC
CCR5	NM_000579	ACCCTCTGGGCCAAGTCAAAGACATTCTGACATCTTAGTATTTGCATATT
CCR6	NM_031409	AGACTTTTGTTATGAGGAGCTGCAGATTAGCTAGGGGACAGCTGGAATTA

Gene Symbol	Accession Number	Sequence
CCR7	NM_001838	GAGCAACATTTTACCCACACACAGATAAAGTTTTCCCTTGAGGAAACAAC
CCR8	NM_005201	GATTGTTGTTATTAACAGCTGATACAGGTATTCTGCTGATGCTACTGCTG
CD24	NM_013230	GCTACTGTGTGTGTGAATGAACACTCTTGCTTTATTCCAGAATGCTGTA
CD36	NM_000072	AATAAACCTCCTTGGCCTGATAGAAATGATCTTACTCAGTGTTGGTGTGG
CDC14A	NM_003672	AGTAATTCAGATGACATTTGAGAAGTAGGGGAAAGGGAATCATGTTGACAG
CDC14B	NM_033332	TAATTTCTTGGTCTCCGAAAACTCAGCTGTGACTGCTTTCCATTAACAGT
CDC20	NM_001255	ATGGACGACATTTGGCCAGTGGTGGTAATGATAACTTGGTCAATGTGT
CDH1	NM_004360	TTCTGTGTAGAGAATGTCACTGTAGTTTTGAGTGTATACATGTGTGGGTG
CDH3	NM_001793	TATTTTCCCTGTTGCGTTGCTATAGATGAAGGGTGAGGACAATCGTGTAT
CDK2AP1	NM_004642	TACTTAGTAGCCGGTTCCCATTTGCCATACAATGTAGGTTCTGCTTAATG
CEACAM6	NM_002483	CATCAGGAGAACATCATAACCCATGAAGGATAAAAGCCCCAAATGGTGGT
CES1	NM_001266	GAGGCCATGAAGGAGCAAGTTTTGTATTTGTGACCTCAGCTTTGGGAATA
CHI3L2	NM_004000	ACCTGGTTTTGTTTTCCTGCAGCTGTTGACTTGTTGCCCTGAAGTACAAT
CHIT1	NM_003465	GTGGGCTTTGATGATGTGGAGAGCTTCAAAACCAAGGTCAGCTATCTGAA
COX6C	NM_004374	GATTTTGAGGAGATGAGGAAGGCTGGTATCTTTCAGAGTGTAAAGTAATC
CP	NM_000096	TGCTATTAATGGAAGAATGTTTGGAAACCTACAAGGCCTCACAATGCACG
CRABP1	NM_004378	TTCCCCTACCAATATTAGGTGATCCCGTTTTCCCCATGACAATGTTGTAG
CRAT	NM_000755	TGTGAGGTTTCACCTCCGTTGCGGCTGTGCTCCTGTGGATAACATTGCTA
CRBPIV	NM_052960	TTATATTGCAGACTGAACAGACGTTTATCTATCCCATTTGGCGACGAGGA
CRYAB	NM_001885	GAAAGTTTCCCCACCAGTGAATGAAAGTCTTGTGACTAGTGCTGAAGCTTA
CSDA	NM_003651	CCTTCACCATCGGCAGGTGACCTAAAGAATTAATGACCATTCAGAAATAA
CTPS	NM_001905	CCAGGATGGTGTTACTGCAGTTGAAGGGCAATATGAAGTTACTTTCTTAA
CX3CL1	NM_002996	GAAAGACTTTTCCAACCCTCATCACCAACGTCTGTGCCATTTTGTATT
CX3CR1	NM_001337	CATCCAATCTAACCACATCAATATTGTCATTCCTGTATTCACCCGTCCAG
CXCL1	NM_001511	CTTGTAAGGCATACTGCCTTGTTTAATGGTAGTTTTACAGTGTTTCTGGC
CXCR4	NM_003467	GTTTTCAGGAGTGGGTTGATTTCAGCACCTACAGTGTACAGTCTTGTATT
CYB5	NM_001914	TGCTTTGGACACGGGAGAAAAGAAGCCATTGCTAACTACTTCAACTGACA
CYB561	AK095244	TTATTTTGCCTGTGGCCAATCTTCTGTGAAATACAATGTGCTGTTGGTGC
CYP2A6	NM_000762	GCTGGGATGAGAGGAAGGAAACCCTTACATTATGCTATGAAGAGTAGTAA
DIP13B	NM_018171	TGCGCGATACAGGTCTAGTTTCGGTAACTGTTCCAGACATCAAGCAATAA
DKFZP761F241	NM_031455	ATTCTCCATAGTATGGTGAAATAGCAGGTGCGTCTTCTAGTTTATTCCTC
DP1	NM_005669	TTCTTCAAGAAAGAATTCCTGCAGCAGTTATTCCCTTACCTGAAGGCTTC
E2F1	NM_005225	GCACTTTGATTTGCTTCCTAACAGCTCTGTTCCCTCCTGCTTTGGTTTTA
EBF	BC038805	ATCTGCAAGTGCAGGGTGTTGGTTAAAGTTGTACCTCCCAAGTATTTG
EBNA1BP2	NM_006824	GCAAGAAAGGGTCAAATAAGAGACCTGGAAAACGAACAAGAGAGAAGATG
EDN1	NM_001955	CTGGCACATTTCAGGGAGAAACTCCAAAGTCCACACAAAGATTTTCTAAG
Ells1	NM_152793	GTTGCCCTTCGTTAGATGCTTCAAACAGTGTAAATCCTATACTGCACCCT
ENPP2	NM_006209	CAGCTACCCAGAAATCCTGACACTCAAGACATACCTGCATACATATGAGA
ENPP5	NM_021572	TGAATAGAAGCAACCAGGCACCATCTCAGCAATGTTTTCTCTTGTTTGTA
EPAC	NM_006105	TTCATGCCCCTTCTTCTCAAAGACATGACCTTCATTCATGAGGGAAACCA
ERBB2	NM_004448	CAGCAATGGTGTCAGTATCCAGGCTTTGTACAGAGTGCTTTTCTGTTTAG
ESR1	NM_000125	GGGATGCTCGAGCACCTGTAAACAATTTTCTCAACCTATTTGATGTTCAAAT
ESR2	NM_001437	ATGAGGGGAAATGCGTAGAAGGAATTCTGGAAATCTTTGACATGCTCCTG
EST	AL080130	TCTCAAGACCAGCTACTTCTGGACAATGGAATACTTTTCAGTCTGGTATG
EST	AK021858	GCTTGCAGTTTGTTTTGGAGATAATACAGTTTCCTGCTATCTGCCGCT
EST	AK092335	CATTCAGAAGTATGGCCAACGGATTTATCTTAAAACCAATGGAGGAAGAAG
EST	AK097484	CCATTTTAGTCTTCATAGCTATTGGCTAAGAGAGATAATGAGCTGATGGTCT

Gene Symbol	Accession Number	Sequence
EST	AK055474	ACTGCTTAACCTAGGCTATGTGAACCCACAGTAAACCTGAAGGTGTTTGA
EST	BC027989	ATCCTTTGGAAACTGGATGTCTTACTGGGTGCTAGAATGAAAATGTAGGT
EST	AK094603	GAAACATCGTTGAGTAGCCTACCTTGATTTCTGTCAAGTTCATAACCAGC
EST	NM_016613	GTAGATGTGTTTTCAGAGCTAGGTACAGAGGAATGTTTGCTACCTTTAG
EST	AK098220	GGTAGGTGTCTCCAGCACTGTATAGGTGTCTGGAATTGAGATAAGAATCA
EST	AI738443	TGGGCTGTTTGACAACCCCATTTTCACCAAGTCAAAACATGGCTTTACCT
EST	AL572847	AGTGCGAAATCCCTTAAACTCATATCAGACCTGTGATGAACAAACTCATACT
EST	AK096401	ATAATACAGAGGATAAATGTTGAGGTGACTGAGACCCCATTTACCCTATG
EST	BQ723065	TATCTATCCCCTCATGTCTTTGTTCTCTATCCCTCGTACACGTTCTCTAT
EST	BU633431	TCTACCTCTAAGGAGATTAAGGAGTGTAAACACAAAGGTGAGGTTGGAGT
F2	NM_000506	TTCTACACACATGTGTTCCGCCTGAAGAAGTGGATACAGAAGGTCATTGA
F3	NM_001993	GCAGCTTCCTAATATGCTTTACAATCTGCACTTTAACTGACTTAAGTGGC
FABP4	NM_001442	GAATTTGACGAAGTCACTGCAGATGACAGGAAAGTCAAGAGCACCATAAC
FABP7	NM_001446	CTTACTTTTGGTGATGTGGTTGCTGTTCGCCACTATGAGAAGGCATAA
FACL2	NM_021122	CAGGGCTTGCACTAAAGAATTGTCATTGTAATAACACTACTTGGTAGCCT
FADS2	NM_004265	AGGCAGAATGAACCCATAGGGAGCTGATCGTAATGTTTATCATGTTACTT
FBP1	NM_000507	TCAAATGCTGTAGAATGCTTGGCACTCCCTAACCAAATGCTGTCTCCATA
FLJ10511	NM_018120	TACCATCATCAGTGAGAGTCTTGAGTTCACTAACATTGTCACCTTCTGGA
FLJ10697	NM_018181	TTCTCTTTCAATGTACCTTCCTTCACCTCGTCGTATATATCCTCGATAAG
FLJ10948	NM_018281	TGGCTAGAAATAATCACGAGGGTTGGGTTTGCTTTGGAAAATGCCTGTCT
FLJ10980	BC040548	CAGATCATCATGCCCATTCATAGCCAGGATTCCTTATCCCCAAAACAGTT
FLJ13322	NM_024722	CACACACTCGGGACGTCTTCATTGAAGATTCACTTACAAAGGAATGTTTC
FLJ14525	NM_032800	ACACACTTTTCAGGAGAAACCTGAGCATGATTTTGGATTCTCCACCTC
FLJ25604	NM_152663	GAAAGGAAAACCAACATGAAACACCAAATAGTGTGTGTGAATCTTCTGGC
FLJ32389	NM_144617	CTACTTCCTTCTGAGTCCTCTACAAAGACATCCGGGTACTACATTTCCAT
FLT1	NM_002019	TTGGATCGCTAAGCTGGCTCTGTTTGATGCTATTTATGCAAGTTAGGGTC
FMO2	NM_001460	AATGGGAAGGAGCCAGAAATGCCATCTTCACCCAGAAACAAAGAATACTG
FMO5	NM_001461	GGGAGCTCAGGTACTCTTTTAGTCATCTTTGTATGTCTTTAGCAGAGTTC
FMOD	NM_002023	AATCCACAAAAGCCAAACCAGCTCATTTCAACAAAGGAGCTCCGATGTGA
FOXA1	NM_004496	TTGGAGGAGAGATAAGTTATAGGGAGCTGGATTTCAAAACGTGGTCCAAG
FTHFD	NM_012190	GGGTCAAGACAGTGACCTTCGAATACTGAAGAAAGGTCTTTGTGAGAAGA
FVT1	NM_002035	TCGGAGCATATATGTAGCTTTTAGGAAAGGCTGATGATGGTATAAAGTTTGC
FXYD3	NM_021910	AGCCATGGGACCTCATATGCAATTTGGGATTTACTAGTAGCCAAAAGGAA
FZD7	NM_003507	ATAGAACTCGGATTCTTTTGCATGATGGGGTAAAGCTTAGCAGAGAATCA
GABRP	NM_014211	GGAGTGCTGGTGAAAAGAGATGAAATGTGGTTGTATGAGCCAATCATAT
GALNT10	NM_017540	TAGCTTTAGAAGTGGCCCTCTATAGAAAGAAGTCAAAAGATGAGGCCC
GATA3	NM_002051	TAGGCGAATCATTTGTTCAAAGCTGTTGGCCCTCTGCAAAGGAAATACCA
GGH	NM_003878	CTTGCTACTCATGGCAAGATTAGGAAGTCACAGATTCTTTTCTATAATGTGC
GLDC	NM_000170	GCAAGTTTGCAGATGTCATAGAGGCTATCCTGGAGACTTAATAGACATT
GNG11	NM_004126	AGGGTTATGTATAAAAGCATATGTGCTACTCATCTTTGCTCACTATGCAGTC
GPCR1	NM_014373	GTCAACTGGAAGTGCTGCTTCATTCCACTTACAATTCCTAATCTTGAGCA
GPD1	NM_005276	TGTGACTCAGGCCTTCCCATCAGGCCTATTTGTCTACCCAATAAAGCGTG
GPX3	NM_002084	GGACAGTGACAACCCTTTCTCTCCAGTTCTCCACTCCAATGATAATAGTT
GRB7	NM_005310	CTTTGTACAGACCGAGAGGCCAGTTGATCTGCTCTGTTTTATACTAGTGA
GRLF1	NM_024342	GAATTCTTGGCTTGTGTCGCATACTGGGTGTCACGGCACACATTTACTCT
GSTM3	NM_000849	AGGATATGTGGAGAATCATCAAGATATGAATTGAATCGCTGCGATACTGG
GSTP1	NM_000852	GAGTACGTGAACCTCCCCATCAATGGCAACGGGAAACAGTGAGGGTTG

Gene Symbol	Accession Number	Sequence
HIS1	NM_006460	CACAAATCAATAAATCAAGGGAGATTATACCAGTAGGACTGAATCAGGGC
HMGCS2	NM_005518	TCAGCTGTTGCCTTCAAGATCCAGATAAACTTTCCTGTCATGTGTTAGAA
HOXB5	NM_002147	GTCTCAGTGATTCGCTTTTGGTATTTGTTTGTAGCTTTCCTGGAAGTCAAAT
HOXB6	NM_156036	TGTCTTAGCTCTGGACGTGAAATACTTCGATGATGATGATGATGATGATG
HRASLS3	NM_007069	GTCTTGTTTTGCTAGAGAGTTTGGGGTTTGGTTTGTGGATTTCATTGTGA
HSD17B4	NM_000414	CTGCTGATACAACAATCATACTTTCAGATGAAGATTTCATGGAGGTGGTC
HSPC163	NM_014184	TGAAGTCACACATGAAAGAAGCCATGATCAAGCTTGGTTTCCACTTGCTC
ID4	NM_001546	CTATAGCTATGTTACGCTAAGCTACTGTCCCATCTCTTGTGATGTGTAAC
IGFBP2	NM_000597	GCACATCCCCAACTGTGACAAGCATGGCCTGTACAACCTCAAACAGTGCA
IGJ	NM_144646	CCAATTATCATGCTCACCTGAAAGAGGTATGCTCTCTTAGGAATACAGTT
IL8RB	NM_001557	TTCTGCAGATAAACAGTAGTGATAGTTGTACCGCAATGTGACTTAATGCC
IMP-1	NM_006546	GTAGATCAGGTTTGCCCACTTGATTGAGAAAGATGTTCCAGTGAGGAA
INPP4B	NM_003866	TGGATTGCATGAGAAGAGAAGGATGCCGCATAGAGAATGTACTGAAGAAT
ITGA7	NM_002206	GACCTTAGTTTGCTGCCATCAGTCTAGTGGTTTCGTGGTTTCGTCTATTTA
ITM2C	NM_030926	AACTGTTTTGAAAGATAACACAGAGGGAAAGGGAGAGCCACCTGGTACTT
KCNK1	NM_002245	TTCTGTACATATGGTTTAGGTCACCAGATCCTAGTGTAGTTCTGAAACTAAG
KIAA0182	XM_050495	ACATGTTTCACACAAGTGTAGAAAATGCCAGGGATCCACCACAAGATGGA
KIAA0239	NM_015288	TAGAGAGAGTTTTGAATTCTACACTGTTGTCTTTCCTCTGTGCTGGCCTA
KIAA0303	AB002301	GGGCGACTGTGTCTTGACTACCTTTCAAAACCAGCACTGTGTGGGAATGT
KIAA0876	AB020683	AGGAAAACAGAGAGAGTGTTACGCAGGAGCAAGCCTTTCATTTCCTTGGT
KIAA1025	XM_034056	GGGAAAAGGGTGGGATTGTCCAGCATGCTTGTATGTATATTTCAGAACCT
KIAA1691	AB051478	TCTCGTCAATGTAATTTCTGTGGTTTCTATTCAGCTTGGGTTTCATGTT
KIF23 (KNSL5)	NM_138555	ACTGACCCAGAGCAAAGCTTTCCCTATGGTTCCAAAGACAACTAGTATTC
KIP2	NM_006383	TGGTGTGCTGTCCACAGATTTGTGAACTCCTGGTAGTAAAACACTTTTGC
KIT	NM_000222	TGTTGGTGTTCCACGTGTTTTATTCCTGTATGTTGTCCAATTGTTGACAG
KRT17	NM_000422	TGCTTCAGTCCCTTCCCCATGCTTCCTTGCCTGATGACAATAAAGCTTGT
KRT5	NM_000424	CAGTTCTATATTCTGCTTCAAATCAGCCTTCAGGTTTCCCACAGCATG
LAD1	NM_005558	ACTCATTTACTCTCAGGTGTAAGAGACTGATGAGACCTTAGAAGCGAATT
LAF4	NM_002285	GGATGTTTCGACTCAAAAGGGACCACGCTGTAAAGTATTCAAAAGCACTA
LAMC2	NM_005562	GACTATGGTGCTGCCTTGCTTCTGTATTTCCTTGGATTTTCCTGAAAGTG
LAPTM4B	NM_018407	TGCAAGCTACATCCTACTGCTTTGAACTTCCAAGTATGTCTAGTCACCTT
LIV-1	NM_012319	ACTAACACAGTTATTCCTATACTGGATTTTAGGTCTCTGAAGAACTGCTGG
LOC157378	BC041379	GGGTAAAGTGTAAGTGCTTTCTTTTGTCCCTAACTTGTGTATTGATGGCA
LOC255743	XM_171089	CACCTCATCAGTATGATTCAGTTTCTCTTATCAATTGGACTCTCCCAGGT
LOC283445	XM_211044	TTGGTTTTGGAGAGAAAGACAAGTTATGAGTAGCTGCTACCCTGGAAC
LOC284665	XM_211581	CCCTGGGTTGAATCAAGATAGTACTTGCAGCTAGATGGATGCTTTTAG
LPL	NM_000237	TGTGGTGCTAACTGTATGTGTCTTTATCAGTGATGGTCTCACAGAGCCAA
LRBA	NM_006726	TCTTTTAAAGTAAATGCAAGTACCAAAGCTCACTACTGCGGTTTGCCTGT
LU	NM_005581	GGCGATGGCCCCATGCTGTCGCTCAGTTCTATCACCTTCGATTCCAATGG
MAP3K8	NM_005204	AACTGATGAATTAGAAGCCATCTGACAGCAGGCCACTAGTGACAGTTTCT
MCCC2	NM_022132	TTAGAGAAATTTCTCTGTGGCTCAGTTTTACCACCCATAAAGCGGAGACA
MCM3	NM_002388	TCCGCCCTACTTCCCATCCCACACAAACCCAATTGTAAATAACATATGAC
MDS029	NM_018464	CACCTTCGCTGGATTCTAAATGTGGTATATTGCAAACTGCAGCTTTCACA
MFGE8	NM_005928	TTCTTAGGCACTGAGGGATCTGAGTAGGTCTGGGATGGACAGGAAAGGG
MGC10500	NM_031477	AGCCAGAAGTACAAAGAGGGGAAGTACATCATTGAACTCAACCACATGA
MGC27171	NM_144591	GGGACTCTATATTGTTGTAGTTCAGGTCTTCATTGACTAAGAGATTGAGAG
MGRN1	AB011116	TCACACATGCTGTCACGCATACACACACGCACATACTCCTGCACATGTTC

Gene Symbol	Accession Number	Sequence
MGRN1	XM_048119	ATTTGTAACTGGTACAAGATGAAGGACAGCAGCTTTCCATCCCTAGTTCAG
MMRN	NM_007351	TTGAGAAACAGCCAGTGTTTTCATTTATCTTTGCTTGCACATCTGCTCTG
MSX2	NM_002449	TGAGAAATACTAGGTACATTCATCCTCACAGATTGCAAAGGTGATTTGGGTG
MT1X	NM_005952	GTGCAGCTGCTGTGCCTGATGCCAGGACAGCTGTGCTCTCAGATGTAAAT
MUC1	NM_002456	GGAGACGCAGTTCAATCAGTATAAAACGGAAGCAGCCTCTCGATATAAC
MYBL2	NM_002466	ATCTTGTCCTGAGGTGTTGAGGGTGTCACGAGCCCATTCTCATGTTTACA
MYC	NM_002467	CTTGGCTGAGTCTTGAGACTGAAAGATTTAGCCATAATGTAAACTGCCTC
NAT1	NM_000662	GAAACAATCTTGTCTATTTGTCATCCAGCTCACCAGTTATCAACTGACGA
NFIB	NM_005596	AGTTTGAACACAAAGGCTCTATGGAAGAAATGCCTCTATGTAGGTGAAGT
NPEPPS	NM_006310	AGAAGTCAGTGGTAACTGCTGCAGGGCTTAATACATTAGTGGTAACTGGT
NPY1R	NM_000909	TTCCGCTTACAATTTGTAGAAACACAAATATCGTTTTCCATACAGCAGTGCC
NQO1	NM_000903	CAGTCCCATGACCTTGGGGTACAATTGTAAACCTAGAGTTTTATCAACTT
NRG1	NM_013957	ACTCAGAGAGTGAAACAGAAGATGAAAGAGTAGGTGAAGATACGCCTTTC
NSEP1	NM_004559	CCAGCAATAAGAAATGAACAAAAGATTGGAGCTGAAGACCTAAAGTGCTTG
PCDH9	NM_020403	TTGCTGTTTAACTGTTTGTTATCATAGTTGGTCAATGCCTGGCAGGTACC
PCOLCE2	NM_013363	AAGCCTTTGCCCTGAGGTGTTACAATCTTGTCTTGCGTTTTCTAAATCAAT
PDGFA	NM_002607	ACCTGTTTTGTATACCTGAGAGCCTGCTATGTTCTTCTTTTGTTGATCCA
PDK3	NM_005391	AAGGACATCTGATATGTGCTGCAACATGGGTGAATCTTGAAGTCATTATG
PGR	NM_000926	ATGTCTGAAGTTATTGCTGCACAATTACCCAAGATATTGGCAGGGATGGT
PLAT	NM_000930	CCCTGGCTCTCAGAAGGTATTCCTTTTGTGTACAGTGTGTAAAGTGTAA
PLIN	NM_002666	GATATATAGACTTTATGTATAGCCACAGTTCATCCCCAACCCTAGTCTTC
PLK	NM_005030	TTGAATTCGGAACTGTCCTTTCCTTGGCTTTATGCACATTAAACAGATGTG
PLOD	NM_000302	ACCATTCACTTTTACTGCTTTGTAGTGACTCGTGCTCTCCAACCTGTCTT
POLR2F	NM_021974	ATCTTCCTGCCCTTGCCCCATGCCCAATTTTCATTCTCACTTTATATGTG
POLYDOM	NM_024500	CCCAGGTTCTCAGCATTGTACATGGCAATAAGCTTGTTCTATCTGGATAC
PON3	NM_000940	TCTGTGGCTTCTGTGTACCATGGGAAAATTCTCATAGGCACCGTATTTCA
PPARBP	NM_004774	AGCTTATAGCTTGTGCTAATACTGTAGCATCAAGCCCAAGCAAATTAGTC
PRAME	NM_006115	TCTGGCTTGGGAAGTACATGTAGGAGTTAATCCCTGTGTAGACTGTTGTA
PRDX4	NM_006406	GACAAAGGAATCCTAAGACAAATTACTCTGAATGATCTTCCTGTGGGTAG
PRKACB	NM_002731	ATTATCGGTGCAGGTAGGTCATTAACACCACTTCTTTTCATCTGTACCAC
PTP4A2	NM_003479	GAAGTATTGCTAGCTGAAGCCAGTTTGACATAGAGAGATGTCAGATTGAT
PTPRK	NM_002844	GTAATTGACTAAAGCATTCCAGTGTCCTCTTTCTAGATTGCCAGCTCATG
PTPRN2	NM_002847	CTACCCCAGAAATAGGACAATTCACTTCATTGACCAGGATGATCACATGG
QDPR	NM_000320	TACAGCAGTCAGAGATGAGTATACTAGAATCATGGATTGCTGGAGGTCTT
RAB31	NM_006868	ACCTTTCCGCCATGTATTCTATTCTGTAGTAAAGCATTTCCATCAACAATGC
RAB5EP	NM_004703	CTCTCAGCAGATTTCAGGTGTAACCATTTGTTAACTGTACTGAAGGTGTG
RALGPS1A	NM_014636	TAAGGACTTTCTCTAATTCTTGTGAATCGTCTCACCCGCAGTAACCACTG
RARRES3	NM_004585	CCCTCTCTCGCTGGCAAAAGTATGATCTAATTGAAACAAGACTGAAGGATCA
RERG	NM_032918	CTGAAAGTACAGTCTACAACTGATAAAACCTTATGATTCTTTTCTCCCC
RGS5	NM_003617	AATCTTTTAGCAACCCCAGTATTCCTTCACTATTTCGCTTCCTGCATTAG
RNPC1	NM_017495	TGGGACAGAGTCAAGGCTCAGAGAATAAAGGTAGCTAATCTCATCATAAT
S100A1	NM_006271	GCCTGTAACAATTTCTTCTGGGAGAACAGTTGAGCAGACAGCCACATTGG
S100A11	NM_005620	TCCTAAGCTTCATGAATACAGAACTAGCTGCCTTCACAAAGAACCAGA
S100A8	NM_002964	GTTGGATATCAACACTGATGGTGCAGTTAACTTCCAGGAGTTCCTCATTC
SAA1	NM_000331	AGCCTACTCTGACATGAGAGAAGCCAATTACATCGGCTCAGACAAATACT
SAA2	NM_030754	CAGGGATTCAAAGTTAGTGAGGTCTATGTCCAGAGAAGCTGAGATATG
SCNN1A	NM_001038	TTGCTGTTGCACCATACTTTCTTGTACATTTGTGTCTCCCTTCCCAACTA

Gene Symbol	Accession Number	Sequence
SCUBE2 (CEGP1)	NM_020974	AGAGATGTTTCCAAGATCGTTCATCCGATTGCTACGTTCCAAAGTGTCCA
SDC2	NM_002998	TTAAGCCTTTGAAGTGCCTCTGATTCTATGTAACTTGTTGCAGACTGGTG
SELENBP1	NM_003944	CATATACATTTACTGACCACTGTTGCTTGTTGCTCACTGTGCTGCTTTTC
SEMA3C	NM_006379	CAGAGTCACAGTTCCCTTTATTTCACATAAGCCCAAACTGATAGACAGTA
SIAH2	NM_005067	CTGGTGTGAAAGACTAAATCAGTAGAGTTGGAAAAGCTTTATAACCGGTG
SIAT4C	NM_006278	TACAACAAGAAGCAGACCATTCACTACTATGAGCAGATCACGCTCAAGTC
SLC40A1 (SLC11A3)	NM_014585	AACCGTTTATATGCACTTTCATGGAGACTGCAATACGTTGCTATGAGCAC
SLC5A6	NM_021095	CTCTGGGAATAGAAGATCCATTTGTCTTTGTGTAGAGCAAGCACGTTTTC
SLC7A5	NM_003486	TAATGACGTGTGTGGCTGGGACCTTCTTTATTCTGTGTTAATGGCTAACC
SLI	AB001451	CCTCCCAGCTGTCGACCCAGATGGGATGTTCGGATCGGTTTGTAATTAA
SLPI	NM_003064	GTTTCCCCTGTGAAAGCTTGATTCCTGCCATATGGAGGAGGCTCTGGAGT
SNCG	NM_003087	AGACCAAGGAGAATGTTGTACAGAGCGTGACCTCAGTGGCCGAGAAGACC
SORBS1	NM_015385	TATGACGAAACAAGAAACAGAGATTTCCAATTGCTCTTTTGTCTTCAGAC
SOX7	NM_031439	GATGCGCAGTCCAGAGCATGTATGAATAATCTCATAAAACAGTATCACAG
SQLE	NM_003129	GCCCTTCTCAGTAGTAGTGCTGTATTGTACAAAGCGTGTTCTGTAATATT
STARD3	NM_006804	TAGGATTATTGAAAGAGTCTGGGACCCTTGTTGGGGAGTGGGTGGCAGGT
STAT6	NM_003153	ACACCCAAAGAGTAAGGCAGATTAGAGACCAGAAAGACCTTGACTACTTC
STK38	NM_007271	GTGTGTTTTGCAAGAATGTGTGGTAAGCCTGGGTAAAGAGAAGGAACT
STK6	NM_003600	ATAGGAACACGTGCTCTACCTCCATTTAGGGATTTGCTTGGGATACAGAA
TCEA3	XM_114075	AATATTTCTTCCCTACTCTTTCCTCATCTCTTCCCAAACTCTCTGCTATTC
TCEAL1	NM_004780	AATCTACAAGTTTCCCTCTTTCAGTCATGAGCCCTACACATTTGCATGAA
TFF3	NM_003226	TCTGCTGAAAGTTCATATCTGGAGCCTGATGTCTTAACGAATAAAGGTCC
TFRC	NM_003234	CCTGAATGAAATATCAGACTAGTGACAAGCTCCTGGTCTTGAGATGTCTT
TGFB2	NM_003238	GAAGGCCTTATTCTACATTTCACCTACTTTGTAAGTGAGAGAGACAAGAAG
TLE3	AB046767	ACCTTTTGGTACCAACATGTTCTGAGGTGTTAGGATTTGGGTTGGGTT
TLE3	NM_005078	GACATTTCAGCGGATGACAAATACATTGTAACAGGCTCTGGTGACAAGAA
TMSB10	NM_021103	CGAGACTGCACGGATTGTTTTAAGAAAATGGCAGACAAACCAGACATG
TNF	NM_000594	GTTGTGTCTGTAATCGCCCTACTATTCAGTGGCGAGAAATAAAGTTTGCT
TNNI2	NM_003282	TGAAGAGGAGAAGTACGACATGGAGGTGAGGGTGCAGAAGACCAGCAAGG
TOP2A	NM_001067	TCTGGCTGCCTCTGAGTCTGAATCTCCCAAAGAGAGAAACCAATTTCTAA
TP53BP2	NM_005426	TTATTGGGCAGTTGGGAAATCAGCTCTCTGTCCTGTTGAGTGTTTTCA
TRAP100	NM_014815	GTGTGTGTGTGTGTGTTTGTGTGTATTTTGTAATATGTGAGGGAAATCTACC
TRIM29	NM_012101	ATTCCCTTAACATGATATAATCCACCCATGCAAATAGCTACTGGCCCAG
UBE2C	NM_007019	GGCGATAAAGGGATTTCTGCCTTCCCTGAATCAGACAACCTTTTCAAATG
VAV3	NM_006113	GACAGAAGTAAATGTTTTCCATATACTTTGTCTTGCCTGTATGCAGCCCT
VGLL1	NM_016267	GCTCAGTTCACTATAAGAAACTATATGTATCTCGTGGATCTGCCAGTACC
VWF	NM_000552	TCAATGAGTGTGTCCGAGTGAAGGAGGAGGTCTTTATACAACAAAGGAA
WWP1	NM_007013	TGCTGCTGGCTTTTCTGAAGACAGGTGCTTGAACTTGTCAGTTTGTTTTA
XBP1	NM_005080	CCAGGTATTGTCTTTTGACATCCAGCAGTCCAAGGTATTGAGACATATTA
YWHAZ	NM_003406	TTGATGATGGGAGGCAGTGAGTCTTGATGATAAGGGTGAGAAACTGAAAT
