# Supplementary material for: Evaluation of MetriGenix custom 4D™ arrays applied for detection of breast cancer subtypes
Source: BMC Cancer. 2006 Mar 15;6:59. doi: 10.1186/1471-2407-6-59 (PMC1421426; doi:10.1186/1471-2407-6-59)
Supplement: Additional File 2 — ANOVA-analysis output. ANOVA analysis of all the genes immobilized on the MetriGenix breast cancer chip. [file 1471-2407-6-59-S2.pdf]

Supplementary table 2
ANOVA Analysis Data Table

Gene symbol	P-Value
FOXA1	4,41E-11
ID4	5,95E-06
TFF3	1,07E-05
SCUBE2 (CEGP1)	6,81E-05
CX3CR1	9,69E-05
XBP1	1,93E-04
CRYAB	4,07E-04
DP1	4,54E-04
GATA3	5,67E-04
CHI3L2	6,24E-04
LOC255743	6,54E-04
FBP1	7,41E-04
CaMKIINalpha	7,66E-04
FLJ10697	8,52E-04
GPCR1	9,06E-04
FLJ10948	0,00105434
GABRP	0,001071564
COX6C	0,001761707
ACTG2	0,001805912
SLPI	0,001839808
PLK	0,001935442
PLAT	0,001975225
ERBB2	0,001997855
ESR1	0,002163017
ASAH1	0,002745665
CYB5	0,002807281
LIV-1	0,003135506
CYB561	0,004314936
EST	0,004765361
CES1	0,004804262
MUC1	0,004851949
RNPC1	0,004857131
KRT17	0,00508879
ANXA8	0,005102396
ANP32E	0,005155266
CCNA2	0,00534309
TCEAL1	0,006267944
MYBL2	0,007314601
WWP1	0,007777517
KIT	0,008176204
B3GNT5	0,008452305
TCEA3	0,009272519
MGC27171	0,009973489
IGFBP2	0,010569537

CCR6	0,012449224
STK6	0,014079383
UBE2C	0,014234206
GRB7	0,014462295
CBR1	0,014697295
SEMA3C	0,015475335
PPARBP	0,01585065
MSX2	0,01759012
Gene symbol	P-Value
QDPR	0,01879313
CCR5	0,018977553
SCNN1A	0,019538218
FLJ10980	0,020524453
HSD17B4	0,020749208
MYC	0,025674744
ESR2	0,0259844
HOXB5	0,027198812
KIF23 (KNSL5)	0,02730503
NAT1	0,02804106
CTPS	0,03242716
ANXA9	0,032539207
TRIM29	0,033407852
FACL2	0,034127403
SOX7	0,03502203
S100A8	0,035610106
RAB5EP	0,037102733
SLC7A5	0,037463706
CHIT1	0,03846853
ACADSB	0,038726125
GALNT10	0,03959366
LAPTM4B	0,04151741
CXCR4	0,047612768
MFGE8	0,048557464
F3	0,048741534
KIAA0303	0,048814416
GNG11	0,0490031
CEACAM6	0,052084636
TOP2A	0,054399602
CDC20	0,055266708
VAV3	0,05532296
PGR	0,06270949
KRT5	0,06407526
CRABP1	0,065156005
RERG	0,06880306
FLJ25604	0,07037382
CX3CL1	0,070794106
APM2	0,07080154
S100A1	0,07158841

HMGCS2	0,073329605
ATP6V1G1	0,078542344
HRASLS3	0,08358025
FZD7	0,08513731
LU	0,08886235
BTG3	0,09270386
PTPRK	0,09331719
ENPP2	0,09390421
CDH3	0,10014556
FMO2	0,10053912
FLT1	0,1034666
BLVRA	0,10629862
POLR2F	0,10639702
RAB31	0,10755934
NQO1	0,108961985
LPL	0,1123477
Gene symbol	P-Value
FMO5	0,11254138
CDK2AP1	0,11868259
TP53BP2	0,119296975
C4A	0,11983251
LAD1	0,12141835
PLOD	0,121677615
ACOX2	0,122658156
STAT6	0,12464152
NFIB	0,1341539
BF	0,1375532
MAP3K8	0,1420296
PON3	0,14249964
MDS029	0,14414787
CCNE1	0,14526032
FXYD3	0,14853236
STARD3	0,15595339
BMP4	0,15693694
AGTR1	0,16030659
RARRES3	0,16113847
SIAT4C	0,16651137
SAA1	0,16762336
ENPP5	0,16963522
FLJ10511	0,17049482
CDC14A	0,17182158
MCM3	0,17348142
CSDA	0,17851794
VWF	0,18030086
FTHFD	0,18781449
ATRCA	0,1949262
BECN1	0,19503096
TRAP100	0,19536301

VGLL1	0,20647576
PTP4A2	0,21010712
C10orf7	0,21072227
GLDC	0,2152065
AOC3	0,21846202
RGS5	0,22463913
APEG1	0,22572647
MCCC2	0,2268849
IL8RB	0,22729664
LOC157378	0,22808915
MT1X	0,22874051
KIAA0876	0,23098443
LRBA	0,23321378
GSTP1	0,25187021
PRDX4	0,2564596
FABP7	0,26275727
INPP4B	0,27627674
STK38	0,28111094
SELENBP1	0,28833914
ITGA7	0,28992188
ATP1A2	0,2900379
LAMC2	0,2912473
ITM2C	0,2914199
ANXA1	0,29300204
Gene symbol	P-Value
GPX3	0,302118
Spacer	0,30415493
NPEPPS	0,30814484
CAV1	0,31017128
SLC40A1 (SLC11A3)	0,3126513
RALGPS1A	0,3131376
SDC2	0,3165861
PRKACB	0,31716362
ADRM1	0,3184579
GPD1	0,3264424
GGH	0,33405292
PRAME	0,33405983
CD36	0,34491867
ALCAM	0,34546727
CDH1	0,34671843
KIP2	0,35152355
FLJ13322	0,35677743
SORBS1	0,36085764
MGRN1	0,36649603
SIAH2	0,37878618
TLE3	0,37895098
CRAT	0,3802302
HOXB6	0,38182795

KCNK1	0,38326746
TGFB2	0,38903442
E2F1	0,39507544
EPAC	0,40071565
AKR1C3	0,40189198
NSEP1	0,40922096
HSPC163	0,4128603
EDN1	0,41534907
CDC14B	0,41694227
TNF	0,42563495
NRG1	0,43956098
FMOD	0,4509794
FLJ14525	0,45127353
IGJ	0,45728353
PDGFA	0,4660699
POLYDOM	0,4686419
DIP13B	0,47639865
FADS2	0,48020712
CCND1	0,49852106
Ells1	0,5031813
TNNI2	0,51737607
DKFZP761F241	0,52240413
TMSB10	0,532218
MMRN	0,5339166
SLI	0,5401665
CP	0,54324913
CCR7	0,54753166
PCDH9	0,55094385
PDK3	0,5623653
PTPRN2	0,5718477
CCR8	0,60700023
ARPC5	0,6098889
Gene symbol	P-Value
EBF	0,6123428
BPHL	0,63477945
TFRC	0,64363045
ALDH1A2	0,6546733
YWHAZ	0,66355926
FABP4	0,6673679
CXCL1	0,6748335
ADRA2A	0,6757176
CD24	0,6846844
PLIN	0,7080254
HIS1	0,7113714
FVT1	0,72638685
SLC5A6	0,7324925
LOC283445	0,74718785
SNCG	0,7610084

F2	0,763065
IMP-1	0,76504534
FLJ32389	0,7697702
PCOLCE2	0,7721332
MGC10500	0,7754117
CYP2A6	0,77591175
APOD	0,78278327
EBNA1BP2	0,7836556
GSTM3	0,7875397
KIAA1691	0,78918236
Thr	0,8009849
KIAA0182	0,80341583
CAPN6	0,8108703
KIAA0239	0,82694614
NPY1R	0,8286034
S100A11	0,8518521
BCL2	0,85512596
KIAA1025	0,87081397
C14orf132	0,87540907
GRLF1	0,8977547
SQLE	0,9155909
CACNA2D2	0,9450517
CRBPIV	0,94613254
CCNE2	0,9477784
LAF4	0,9525647
LOC284665	0,98907566
