# Supplementary figures and images for: Evaluation of MetriGenix custom 4D™ arrays applied for detection of breast cancer subtypes
Source: BMC Cancer. 2006 Mar 15;6:59. doi: 10.1186/1471-2407-6-59 (PMC1421426; doi:10.1186/1471-2407-6-59)

Supplementary figure S1  
226 “intrinsic” gene cluster diagram

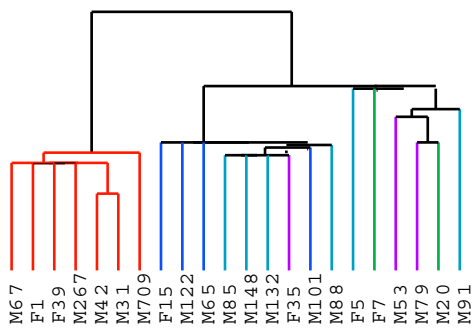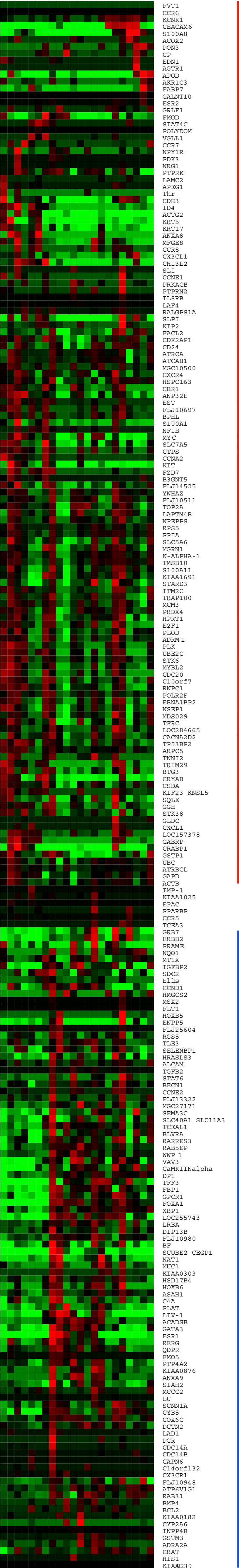

Supplement: Additional File 3 — Hierarchical clustering of "intrinsic" only genes. Hierarchical clustering diagram of the 226 "intrinsic" only gene subset immobilized on the MetriGenix chip. Coloured branches represent the different subtypes as previously determined using a different DNA microarray platform [10]; Dark blue = luminal A, light blue = luminal B, green = normal-like, red = basal-like and purple = ERBB2+. M = early breast cancer, F = locally advanced breast cancer. [file 1471-2407-6-59-S3.pdf]
