# Supplementary material for: Evaluation of MetriGenix custom 4D™ arrays applied for detection of breast cancer subtypes
Source: BMC Cancer. 2006 Mar 15;6:59. doi: 10.1186/1471-2407-6-59 (PMC1421426; doi:10.1186/1471-2407-6-59)
Supplement: Additional File 4 — PAM-analysis output. PAM output of the most differentially expressed genes between the two groups: luminal and non-luminal. [file 1471-2407-6-59-S4.pdf]

Supplementary table 3
PAM output genes

PAM-results		
**Gene Symbol**	**1 score (luminal)**	**2 score (non-luminal)**
SLPI	-0,2973	0,2478
GATA3	0,2374	-0,1978
FLJ1697	-0,1801	0,1501
ESR1	0,176	-0,1466
LOC255743	0,1638	-0,1365
CRYAB	-0,1408	0,1173
IGFBP2	0,1144	-0,0953
LIV-1	0,0923	-0,0769
CHI3L2	-0,0905	0,0754
ATRCA	0,0836	-0,0697
CDH3	-0,0789	0,0657
CDK2AP1	-0,0767	0,0639
TFF3	0,0735	-0,0612
SAA1	-0,0603	0,0503
FOXA1	0,0511	-0,0426
TCEAL1	0,0422	-0,0352
S1A8	-0,0416	0,0347
MFGE8	-0,0395	0,0329
HRASLS3	0,0372	-0,031
ID4	-0,0365	0,0304
ACADSB	0,0364	-0,0303
NAT1	0,0321	-0,0268
GABRP	-0,0314	0,0261
FBP1	0,0121	-0,0101
TRIM29	-0,0084	0,007
CX3CL1	-0,0076	0,0063
KRT5	-0,0039	0,0032
